# Muriated Tincture of Iron in Fluor Albus

**Published:** 1876-02

**Authors:** John M. Johnson

**Affiliations:** Atlanta, Ga.


### Muriated Tincture of Iron in Fluor Albus—Mode of Application—Wool Pessaries, etc.

By JOHN M. JOHNSON, M.D., of Atlanta, Ga.

Since 1844, I have rarely ever used any other remedy for Fluor Albus, both vaginal and uterine, than the muriated tincture of iron, diluted with water and injected into the vagina with a female syringe.

My manner of doing it is to place the patient in bed and on her back; elevate her hips with a small pillow; take of muriated tincture of iron one part, water two parts; fill an ounce female syringe, and pass the point of the syringe down towards the rectum, so as to bring the fluid in contact with the neck of the womb, and discharge it slowly, withdrawing the instrument by littles, so as to keep the entire walls of the vagina in contact with the iron and water as long as possible, and cause the retention of the wash. This I repeat every night and morning for three days and nights, and then every night only, until the diseased surface sloughs off, which will require from eight to twelve days. When done, I heal the surface with flax-seed tea, which will require two days more; and the cure, under ordinary circumstances, will be complete.

Sometimes greater strength than one-third is required, and I have even been obliged to employ the full strength. When I fail to bring away the yellow strips of epithelium in large quantities, I increase the strength until I do. Sometimes the soreness is very great; so great that it confines the patient to bed for several days; but so much the better, for the cure is only the more certain. In such cases I introduce a poultice of ground flax-seed or slippery elm, warm or cold, as the case may require. This I introduce through a speculum, forcing it through with a crammer, until the vagina is full. It is easily washed out with a Davidson syringe when desirable to do so.

The physiological section is: first, the removal of the epithelium; second, a powerful stimulating and constringing effect upon the diseased mucous surface and follicles. When this is cured by the poultices or flax-seed tea, in cases where much inflammation has been induced, the disease will be found to have disappeared. Ordinarily, no application, such as poultices or washes, will be necessary.

This remedy will also be found to have great efficacy in diseases of the inner surfaces of the neck and womb, as endometritis, diseases of the inner os, and the cervical canal. The womb undoubtedly has a function that has scarcely been discussed. I mean that of suction—the faculty of drawing up into the internal cavity whatever stimulates or irritates the neck. In my opinion the spermatazoa enter the womb by this physiological action, and other substances enter it by the same law. The womb and neck are made of erectile tissues, and the canal is lined with ciliary epithelium; not certainly to aid in the descent of the mucus and other fluids secreted or flowing into the vagina from above, but to aid the ascent of matters necessary to the complete functions of the organ. If, therefore, the hips are elevated and the dilute tincture of iron left in contact with the neck, the first effect is to stimulate and constringe; the second is to tolerate the presence of the fluid as though it was not in contact, and then the process of sucking up begins.

Sometimes there is a relaxed and phagedenic condition of the vagina and neck, and in such cases, in addition to flour albus, there is prolapsus also. When this is the case, I put the patient in the knee-and-elbow position, and bend the back downward; open the mouth of the vagina with a Sims' speculum; let the womb go down (up if you choose), and after bringing the part fully to view, then saturate some well-carded and well-washed wool rolls* with the dilute tincture of iron, and introduce them, packing the vagina full, removing and renewing every twenty-four hours, until the sloughing heretofore described takes place; then heal by the poultices, etc., recommended above; and when this is done, continue the wool, taking care to so introduce it with forceps as to keep the neck straight, and when well and carefully and properly filled, secure it, and support the perineum by a strip between the legs, attached behind and before to a belt around the waist, secured by suspenders above to keep everything steady, and a little wool pad one and a half inches thick, two inches long, and one and a half wide, fastened to the strip between the legs to support the perineum.* This done you will only have to renew your wool pessary every twenty-four hours, to secure the perfect relief of your patient in a short time. All the mal-conditions of the womb can be relieved in this way, except deformities from loss of substance. Care must be taken to keep the iron off the bed and other clothing, as the stains cannot easily be removed.

